# Addictive Internet Use among Korean Adolescents: A National Survey

**DOI:** 10.1371/journal.pone.0087819

**Published:** 2014-02-05

**Authors:** Jongho Heo, Juhwan Oh, S. V. Subramanian, Yoon Kim, Ichiro Kawachi

**Affiliations:** 1 Public Health Joint Doctoral Program, San Diego State University & University of California, San Diego, La Jolla, California, United States of America; 2 Department of Medicine, Seoul National University College of Medicine, Seoul, Korea; 3 Department of Social and Behavioral Science, Harvard School of Public Health, Boston, Massachusetts, United States of America; 4 Department of Health Policy and Management, Seoul National University College of Medicine, Seoul, Korea; Catholic University of Sacred Heart of Rome, Italy

## Abstract

**Background:**

A psychological disorder called ‘Internet addiction’ has newly emerged along with a dramatic increase of worldwide Internet use. However, few studies have used population-level samples nor taken into account contextual factors on Internet addiction.

**Methods and Findings:**

We identified 57,857 middle and high school students (13–18 year olds) from a Korean nationally representative survey, which was surveyed in 2009. To identify associated factors with addictive Internet use, two-level multilevel regression models were fitted with individual-level responses (1st level) nested within schools (2nd level) to estimate associations of individual and school characteristics simultaneously. Gender differences of addictive Internet use were estimated with the regression model stratified by gender. Significant associations were found between addictive Internet use and school grade, parental education, alcohol use, tobacco use, and substance use. Female students in girls' schools were more likely to use Internet addictively than those in coeducational schools. Our results also revealed significant gender differences of addictive Internet use in its associated individual- and school-level factors.

**Conclusions:**

Our results suggest that multilevel risk factors along with gender differences should be considered to protect adolescents from addictive Internet use.

## Introduction

Internet use is recognized as an essential part of modern life. Owing to web-based technologies and increases of Internet access in Latin America and Asia, Internet use has increased dramatically across the world reaching the number of global Internet users more than 2.3 billion in 2011 [1].

On the other side of this popularity, a new psychological disorder has emerged: “Internet addiction”, also inconsistently referred to as “excessive Internet use” [2], [3], “problematic Internet use” [4], [5], “Internet dependency” [6], [7], or “pathological Internet use” [8], [9]. Such discrepancy is largely attributable to lack of consensus in definitions across studies that focused on different symptoms of Internet addiction. Young [3] defined Internet addiction as “maladaptive pattern of Internet use leading to clinically significant impairment or distress”. Kandell [10] later defined it as “a psychological dependence on the Internet, regardless of the type of activity once logged on” [11]. Other studies have even not given it a clear definition. To measure or diagnose these addictive symptoms related with Internet use, some studies have developed their own assessment tools. Most of the Internet addiction studies developed measures based on the Diagnostic and Statistical Manual of Mental Disorders (DSM) criteria [11]. Young [3] developed the 8-question diagnostic Questionnaire with modification of the criteria for compulsive gambling (DSM-IV). Morahan-Martin and Schumacher [8] later developed the Pathological Internet Use scale of 13-questions by reconstructing the DSM-IV criteria. More recent studies developed new measures independently with DSM criteria. Using factor analysis methods, Caplan [12] and Widyanto and Mcmurran [13] created their own measures. Tao et al. [14] developed their measure using item-response theory. These variations in the definitions and measures have fueled controversies over inclusion of Internet addiction in the DSM [15], [16].

Despite the lack of consensus on its definition and measurement, evidence of Internet addiction has accumulated since the mid-1990s. Case and empirical studies revealed that Internet addiction was characterized by adverse effects on the individual's psychological well-being [17], [18], academic failure [17], [19], reduced work performance [20] or job loss [21], sleep deprivation [22], social withdrawal [21], [23], little or no self-confidence [21], [24], poor diet [20], [25], family problems [21], [25], marital breakdown [21], and even violence associated with blocked access to online games [26] or cardiopulmonary-related death from excessive use [27], [28].

However, these studies have some limitations. First and most critically, a majority of the research suffered from sampling bias due to convenience sampling and small sample size as they recruited subjects through the Internet [3], [13], [24], [29]–[32]. Inevitably, this sampling of self-selected participants caused mixed or contradicted results between the studies. Second, though the effects of environmental factors on addictive behaviors have been well established [33], [34], most of the past papers on Internet addiction have focused primarily on associations with individual personality such as low self-esteem [24], loneliness [8], low self-disclosure or anti-social behavior [35], stronger suicidal intention [36], and sensation-seeking [6], [7], [24]. Specifically, no empirical studies examined the associations with family factors (e.g. family income or parental educational attainment) and school environmental factors though it is well known that parental socioeconomic status (SES) and school characteristics were associated with risks of adolescents' addictive behaviors [37]–[39]. Last, despite past studies having consistently reported higher risks of Internet addiction among boys [40], [41], few studies have identified gender differences in Internet addiction.

To fill these gaps in past studies with social epidemiological perspectives, we examine the individual- and contextual-level correlates of Internet addiction with a multilevel statistical method using nationally representative survey data of South Korean adolescents. Due to higher prevalence of Internet addiction in Korean adolescents than adults [42], we focus on Internet addiction among adolescents. This study also examines gender differences in Internet addiction among the population.

South Korea is one of the most highly digitalized societies in the world. The Internet penetration rate in South Korea exceeded 75 percent in 2011 [1]. More than half of the 50s age-group and almost 100% of teenagers are using the Internet in their daily life [43]. After a series of crimes and death related to Internet addiction, South Korea has regarded Internet addiction as a social and public health problem. The government initially developed the Korean-version of the Internet addiction measurement scale (KS-scale) and has introduced into middle and high schools for screening addictive Internet users [44]. Moreover, to curb excessive online gaming among adolescents, the government implemented compulsive policies called “Internet Shutdown” and “Cooling Off” in 2011 and 2012 respectively to limit adolescents' online gaming at midnight and amounts of time spent for online games [45]. A nation-wide survey specified to Internet addiction in 2010 showed that 8.0% in the whole population were addicted to Internet; 12.4% of adolescents were using Internet addictively [42]. Given that Internet users have been increasing exponentially around the world especially with the popularity of social network services (SNS), this study could provide information to prevent and intervene in adolescent Internet addiction for other countries where it has not emerged yet as a social and public health issue.

We are interested in answering the following questions: 1) Is higher parental SES inversely correlated with adolescents' addictive internet use? 2) Are school contexts associated with adolescents' addictive internet use regardless of individual-level factors? 3) Are these associations of individual- and school-level factors different between genders?

## Methods

### Source of Data

Out of 75,066 samples from the Fifth Korean Youth Risk Behavior Web-based Survey (KYRBWS) conducted in 2009, we identified 57,857 students from 400 middle and 400 high schools after dropping samples missing values for parental education level. The KYRBWS is a nationally representative survey producing annual data to monitor adolescent (13–18 year olds) health behaviors. The KYRBWS was produced by the Korea Centers for Disease Control and Prevention (KCDC) and approved by the ethics committees of the KCDC. The written informed consent was obtained from each student's parents for the survey. To have a nationally representative sample, the survey used the stratified two-stage random cluster sampling method. A total of 800 middle and high schools (primary sampling units) were selected via random sampling from each stratum of 135 strata that were identified using administrative districts and characteristics of schools Then, one class (secondary sampling units) in each school grade was randomly sampled from each selected school. All the students of the sampled classes were requested to complete an anonymous web-based survey during an hour of their regular class time in a computer room of each selected school. Survey objectives and the entire survey process were explained to the students before the survey was conducted. The students were required to log into the KYRBWS website with a randomly assigned number and complete the self-administered questionnaire. The overall response rate of the Fifth KYRBWS study was 97.6%.

### Measurement

Internet addiction was assessed by the simplified Korean Internet Addiction Self-assessment Tool (KS scale) (see Table S1), which was developed by the Korean government and used nation-wide in Korea with a definition of “having trouble in one's daily life due to withdrawal and tolerance in Internet use regardless of devices” [44]. The test for reliability and construct validity of the scale is described in more detail elsewhere [44]. This official measure has been adopted for nationwide Internet addiction screening and annual surveillance among Korean adolescents [42]. The scale consisted of 20 questions inquiring about 6 domains: disturbance of adaptive functions, positive anticipation, withdrawal, virtual interpersonal relationship, deviant behaviors, and tolerance. Responses were scaled with 4 categories from “never” to “always yes”. In this study, rather than adopting the measurement itself that has cut-points of three categories (addiction, latent addiction, and normal), we measured the severity of Internet addiction with a continuous variable by summation of each response [from 1 (never) to 4 (always yes)] with a range from 20 to 80. We treated this score of addictive Internet use as an outcome variable in the study.

As shown in table 1, key individual-level variables used in the analysis included demographic characteristics; self-rated academic achievement; parental socioeconomic status (SES); tobacco, alcohol, and substance use; and physical activities and psychological status. Self-rated academic achievement was a five-level categorized variable from very high to very low. We treated self-rated academic achievement as a continuous variable in the main analysis. Parental SES was measured by parent's educational attainment and the Family Affluence Scale (FAS) [46]. Paternal and maternal educational attainment were categorized in three levels (middle school-or-less, high school, and college-or-higher). The FAS was measured by summation of answers of four items: 1) having one's own bedroom (yes = 1, no = 0); 2) frequency of family trips per year; 3) the number of computers at home; and 4) the number of vehicles owned by family. Tobacco and alcohol use were measured by the average number of cigarettes and average volume of alcohol consumed in the past 30 days. Substance use was categorized into three levels: never, past use, and current use. Categories of physical activity were strenuous exercise, moderate exercise, and weight training, which were estimated by the number of days of exercise over 30 minutes, 20 minutes, and days of weight training, respectively. Of psychological factors, self-rated sleep satisfaction was scaled into five categories from very good to very poor. Depressive symptoms and suicidal ideation were dichotomized as yes or no to questions whether the student has ever had depressed moods or suicidal ideation in the past twelve months. We included two types of school-level variables: the urbanicity of the school's location (metropolitan, urban, and rural) and school type by gender mix (boys', girls', and co-educational).

**Table 1:** Characteristics of Korean adolescents.

Variables	Responses	N	%
Gender	Male	39,612	52.8
	Female	35,454	47.2
School grade	Middle school 1st	12,714	17.0
	Middle school 2nd	12,868	17.1
	Middle school 3rd	12,827	17.1
	High school 1st	12,477	16.6
	High school 2nd	12,427	16.6
	High school 3rd	11,753	15.7
Self-rated academic	Low	9,714	12.9
achievement	Middle low	19,138	25.5
	Middle	20,219	26.9
	Middle high	17,583	23.4
	High	8,412	11.2
Paternal education	≤Middle school	5,190	6.9
	= High school	28,209	37.6
	≥College	29,049	38.7
	Unknown	9,124	12.2
	Non-response	3,494	4.7
Maternal education	≤Middle school	4,937	6.6
	= High school	35,957	47.9
	≥College	21,044	28.0
	Unknown	9,766	13.0
	Non-response	3,362	4.5
Substance use	Never	74,569	99.3
	Past use	267	0.4
	Current use	230	0.3
Depressive symptoms	Yes	28,273	37.7
	No	46,793	62.3
Suicidal ideation	Yes	14,458	19.3
	No	60,608	80.7
Location of schools	Metropolitan	39,287	52.3
	Urban	26,407	35.2
	Rural	9,372	12.5
School type	Coeducation	47,429	63.2
	Girls'	13,500	18.0
	Boys'	14,137	18.8
	Range	Mean	SD
Family Affluent Scale	0–9	4.52	1.8
Alcohol use	0–5	0.50	1.2
Tobacco use	0–6	0.42	1.2
Strenuous exercise	0–5	1.81	1.7
Moderate exercise	0–5	1.77	1.6
Weight training	0–5	1.26	1.6
Self-rated sleep satisfaction	1–5	2.80	1.2

### Statistical Analysis

A two-level, random intercept multilevel regression model was fitted with individuals (level 1) nested within schools (level 2) to estimate the associations of individual determinants and school context simultaneously using *MLwiN* (development version 2.22). Chow test was applied to detect significant gender differences in terms of slopes and intercepts between the stratified regressions [47] that were fitted separately to boys and girls. We obtained maximum-likelihood estimates by Iterative Generalised Least Squares (IGLS), and then switched to Markov Chain Monte Carlo (MCMC) function. The MCMC was conducted to burn-in for 500 simulations for starting values of the distribution to discard, and was followed by 5,000 further simulations to get the precise estimate and distribution of interest. Once convergence diagnostics were confirmed, the simulated values and 95% credible intervals (CI) were obtained.

## Results

Table 2 shows students' primary and secondary purposes for Internet use aside from academic purposes, according to gender in the middle and high schools. Regardless of the school, boys' primary and secondary purpose of Internet use were online gaming and information searching, respectively. Girls reported blogging and updating a personal homepage, searching for information, and using messengers and chatting as their primary and secondary purposes.

**Table 2:** Primary and secondary purposes of Internet use (except for academic purposes) by gender in middle and high schools.

*Male*		
Purpose	Middle school	High school
Primary (%)	Online gaming (67.0)	Online gaming (44.8)
Secondary (%)	Searching information (11.2)	Searching information (21.3)
*Female*		
Purpose	Middle school	High school
Primary (%)	Blogging/Updating personal homepages (23.0)	Searching information (23.9)
Secondary (%)	Chatting/Using messengers (20.2)	Blogging/Updating personal homepages (22.1)

Table 3 presents the result of multilevel regression modeling to predict addictive Internet use among adolescents. Girls were much less likely to be addicted to the Internet than boys. The score of addictive Internet use increased gradually during middle school years, yet they decreased during high school years. Self-rated academic achievement was inversely associated with addictive Internet use. As the parental education level and the FAS increased, the score of addictive Internet use was significantly decreased. Tobacco use was inversely associated with addictive Internet use while alcohol use was not a significant factor. Substance use showed the strongest association with addictive Internet use. All the variables of physical activities showed inverse associations with addictive Internet use. Higher scores of addictive Internet use were associated with higher levels of sleep dissatisfaction. Psychological characteristics such as depressive symptoms and suicidal ideation showed positive associations with addictive Internet use. Regarding school characteristics, girls attending girls' schools were more likely to have addictive Internet use than those attending coeducational schools.

**Table 3:** Multilevel regression estimates (along with their SE) based on a two-level model for the extent of addictive Internet use among Korean adolescents.

	Estimate	S.E.	CI (2.5% 97.5%)
*Fixed Parameters*			
Constant	31.7	0.33	(31.07 32.35)
Gender (vs. Male)	−4.41	0.10	(−4.61 −4.21)
School grade (vs. Middle school 1st)			
Middle school 2nd	1.35	0.13	(1.09 1.62)
Middle school 3rd	1.53	0.13	(1.27 1.79)
High school 1st	0.83	0.16	(0.54 1.15)
High school 2nd	0.69	0.16	(0.38 1.00)
High school 3rd	−0.06	0.16	(−0.39 0.26)
Self-rated academic achievement	−0.38	0.03	(−0.45 −0.32)
Paternal education (vs. ≤ Middle school)			
= High school	−0.39	0.16	(−0.71 −0.08)
≥ College	−0.44	0.17	(−0.79 −0.11)
Maternal education (vs. ≤ Middle school)			
= High school	−0.20	0.16	(−0.50 0.12)
≥ College	−0.66	0.18	(−1.00 −0.31)
Family Affluent Scale	−0.08	0.02	(−0.1 −0.03)
Alcohol use	0.02	0.04	(−0.06 0.09)
Tobacco use	−0.26	0.04	(−0.33 −0.18)
Substance use (vs. Never)			
Past use	2.90	0.76	(1.45 4.39)
Current use	7.82	0.98	(5.90 9.76)
Strenuous exercise	−0.26	0.03	(−0.32 −0.20)
Moderate exercise	−0.12	0.03	(−0.18 −0.07)
Weight training	−0.30	0.03	(−0.35 −0.24)
Self-rated sleep satisfaction	−0.72	0.03	(−0.79 −0.66)
Depressive symptoms (vs. No)			
Yes	1.65	0.09	(1.48 1.81)
Suicidal ideation (vs. No)			
Yes	2.25	0.11	(2.04 2.50)
Location of schools (vs. Rural)			
Metropolis	−0.34	0.17	(−0.66 0.01)
Urban	−0.29	0.18	(−0.63 0.06)
School type (vs. Coeducation)			
Boys'	0.26	0.15	(−0.03 0.56)
Girls'	0.52	0.15	(0.22 0.82)
*Random Parameters*			
School level	1.23	0.12	(1.01 1.48)
Individual level	78.84	0.47	(77.94 79.8)
Units: School	800		
Units: individual	57,857		

With confirmation of the Chow test [F (17, 57,823) = 163.62, p<0.001], gender stratified analysis revealed different patterns of associations between boys versus girls across all the variables (Table 4). The association of poor self-rated academic achievement with addictive Internet use was stronger in boys than in girls. Parental educational status was inversely associated with addictive Internet use among boys while showing no association among girls. Tobacco and alcohol use showed the opposite associations between boys and girls: 1) a statistically significant association between drinking and addictive Internet use in girls, yet non-significant in boys; 2) a significant association between smoking less and addictive Internet use in boys but not in girls. Boys who reported substance use at the time of survey had much higher risk of addictive Internet use compared to girls. The associations of addictive Internet use with physical activities and psychological characteristics were stronger in boys than girls. With respect to school context variables, girls' schools had a positive association with addictive Internet use; whereas, boys' schools had no association. Urbanicity of school locations showed no correlation with addictive Internet use.

**Table 4:** Multilevel regression estimates (along with their SE) based on a gender-stratified two-level model for the extent of addictive Internet use among Korean adolescents.

	Boys	S.E.	CI (2.5% 97.5%)	Girls	S.E.	CI (2.5% 97.5%)
*Fixed Parameters*						
Constant	32.89	0.51	(31.89 33.87)	26.00	0.40	(25.21 26.78)
School grade (vs. Middle school 1st)						
Middle school 2nd	1.54	0.21	(1.14 1.97)	1.11	0.18	(0.77 1.46)
Middle school 3rd	1.72	0.20	(1.32 2.12)	1.34	0.17	(1.01 1.68)
High school 1st	1.02	0.23	(0.57 1.48)	0.66	0.20	(0.28 1.05)
High school 2nd	1.05	0.24	(0.57 1.52)	0.35	0.20	(−0.02 0.73)
High school 3rd	0.22	0.25	(−0.27 0.70)	−0.30	0.19	(−0.60 0.14)
Self-rated academic achievement	−0.58	0.05	(−0.68 −0.49)	−0.16	0.04	(−0.24 −0.08)
Paternal education (vs. ≤ Middle school)						
= High school	−0.60	0.25	(−1.09 −0.12)	−0.16	0.20	(−0.55 0.23)
≥College	−0.90	0.27	(−1.41 −0.38)	0.02	0.22	(−0.41 0.43)
Maternal education (vs. ≤ Middle school)						
= High school	−0.19	0.25	(−0.67 0.30)	−0.23	0.20	(−0.61 0.16)
≥College	−1.02	0.28	(−1.56 −0.49)	−0.32	0.22	(−0.75 0.12)
Family Affluent Scale	−0.09	0.03	(−0.15 −0.02)	−0.07	0.03	(−0.13 −0.01)
Alcohol use	−0.05	0.06	(−0.16 0.06)	0.14	0.06	(0.03 0.25)
Tobacco use	−0.44	0.05	(−0.54 −0.34)	0.12	0.06	(0.00 0.25)
Substance use (vs. Never)						
Past use	2.30	1.06	(0.25 4.37)	3.44	1.09	(1.34 5.55)
Current use	8.90	1.26	(6.41 11.36)	4.58	1.62	(1.44 7.86)
Strenuous exercise	−0.29	0.04	(−0.38 −0.21)	−0.20	0.04	(−0.28 −0.12)
Moderate exercise	−0.18	0.04	(−0.27 −0.09)	−0.05	0.04	(−0.13 0.03)
Weight training	−0.32	0.04	(−0.38 −0.24)	−0.26	0.04	(−0.34 −0.17)
Self-rated sleep satisfaction	−0.82	0.05	(−0.92 −0.72)	−0.62	0.04	(−0.71 −0.54)
Depressive symptoms (vs. No)						
Yes	1.76	0.14	(1.49 2.02)	1.55	0.11	(1.34 1.76)
Suicidal ideation (vs. No)						
Yes	2.45	0.18	(2.10 2.80)	2.09	0.13	(1.84 2.33)
Location of schools (vs. Rural)						
Metropolis	−0.33	0.26	(−0.83 0.16)	−0.32	0.19	(−0.68 0.06)
Urban	−0.28	0.27	(−0.80 0.26)	−0.32	0.19	(−0.70 0.06)
School type (vs. Coeducation)						
Boys'	0.21	0.19	(−0.15 0.57)			
Girls'				0.68	0.13	(0.43 0.94)
*Random Parameters*						
School level	1.99	0.25	(1.55 2.50)	0.79	0.12	(0.57 1.05)
Individual level	94.52	0.77	(93.03 96.04)	61.65	0.52	(60.67 62.69)
Units: School	658			647		
Units: individual	29,492			28,365		

## Discussion

To our knowledge, this is the first study that examined associations of addictive Internet use with individual-level factors and school-level environmental factors using multilevel analysis with a nationally representative sample. Our novel finding is that there were associations between the adolescents' addictive Internet use and school contexts even after controlling for individual-level characteristics: girls in girls' schools were more likely to be addicted to the Internet than those in coeducational schools. Additionally, we found gender differences in addictive Internet use from the gender stratified analysis: 1) lower parental educational attainment was associated only with boys' addictive Internet use, and 2) alcohol use was a risk factor of addictive Internet use for girls only; whereas, smoking is a risk factor for boys only.

First, our hierarchical regression analysis showed that girls in girls' schools were more likely to be addicted to the Internet compared with girls in coeducational schools after controlling for individual-level factors. The contexts of girls' schools may contribute to girls' addictive Internet use with fostering their online networking based on abundant offline same-sex networks within their schools. Korean students in single gender schools seemed to have more same-sex friends than those in coeducational schools because they spend most of their time in school in pursuit of academic excellence, and making opposite gender friends is usually not welcomed by parents concerned about their children's academic achievement [48]. Given that girls have a greater tendency to cherish interpersonal relationships in offline networks and are generally more cautious in creating new relationships online [48]–[50], they may take advantage of cyberspace to maintain relationships and reinforce their own identities through communicating and sharing information on their common interests via instant messaging, chatting, and visiting friends' personal websites [10], [48], [51]. Some girls could also make boyfriends online or offline; however, it might not contribute to Internet addiction as they might want to spend more time face-to-face. Boys in boys' schools also might tend towards Internet addiction based on their relatively abundant offline networks within the schools via online gaming together. However, as shown in the results, school type was not a significant factor for boys' addictive Internet use perhaps because online gaming networks are usually established nationwide or worldwide [52].

**Figure 1:**
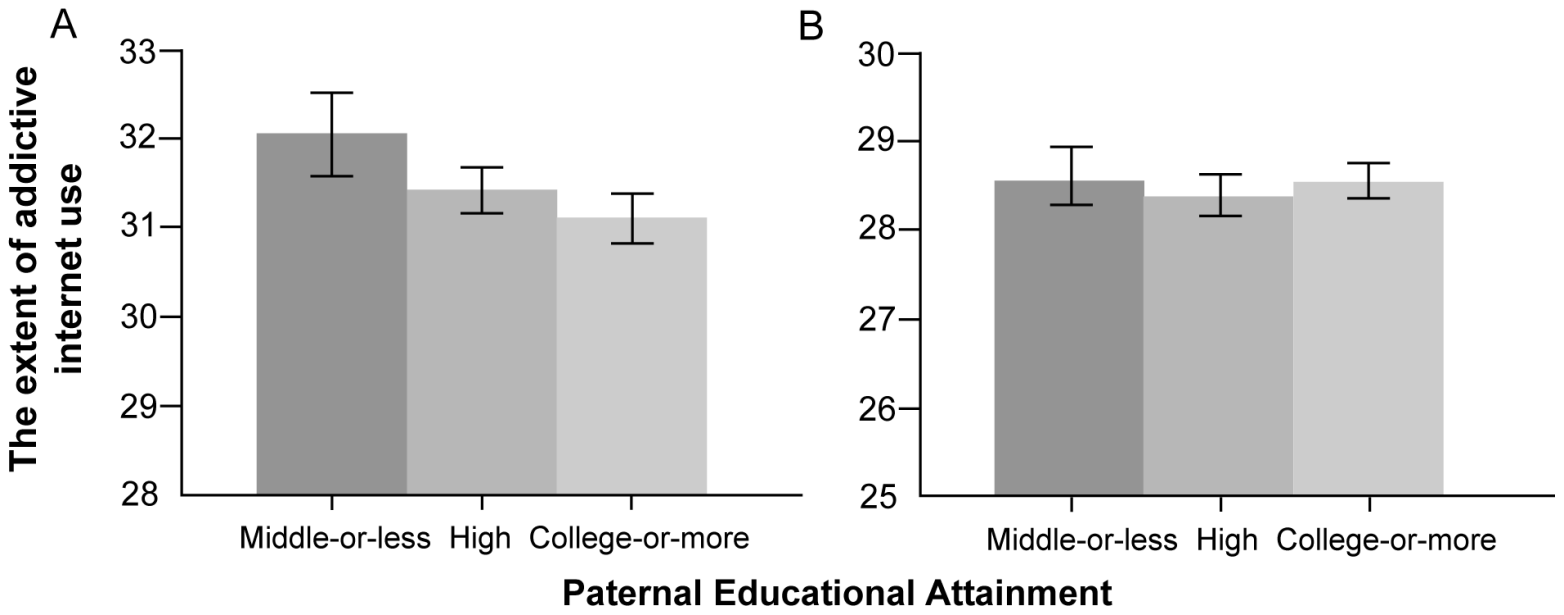
The extents of addictive internet use of Korean boys (A) and girls (B) across paternal education.

**Figure 2:**
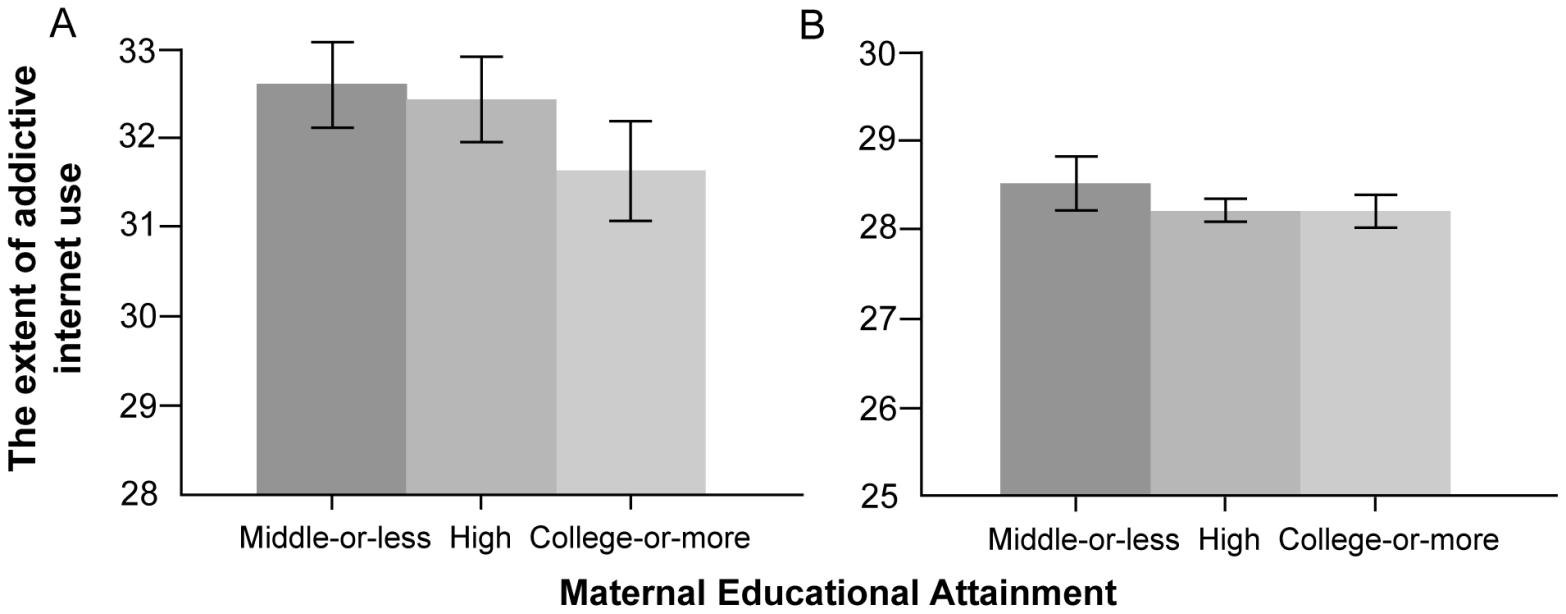
The extents of addictive internet use of Korean boys (A) and girls (B) across maternal education.

Another novel finding in our study is that parental SES was inversely associated with adolescents' addictive Internet use. Parents of higher education attainment might be able to guide their children toward desirable Internet use and supervise children's Internet use effectively based on their knowledge of the Internet and its devices. Moreover, adolescents whose parents had higher SES might use the Internet less addictively due to their higher self-esteems [53]. Notably, gender stratification showed that a higher parental educational level was only significantly associated a lower score of addictive Internet use in boys (Figure 1-A and 2-A). This could be explained by parents' supervision focused on their boys. Korean parents usually had concerns on their boys' Internet use because they were more accessible and vulnerable to addictive online games and sexual/violent images [51].

We also found several other variables associated with addictive Internet use among both genders, yet their directions and magnitudes were varied in gender stratification. In the high school grades, the addictive Internet use score was decreased. This is contrasted with past studies that reported no association between age and Internet addiction [9], [54]. This inconsistency seems to lie in the difference of sampling methods or academic and cultural contexts (Taiwan vs. European countries vs. Korea). Higher pressure for academic achievement of in the Korean society might limit high school students' online networking and/or time spent for online gaming [48].

Of cigarette smoking and alcohol drinking, our results showed an inverse association of addictive Internet use with smoking and an insignificant association with drinking; however, gender stratification showed complex patterns in the associations of addictive Internet use with drinking and smoking. Drinking and smoking seemed to be complementary for girls' addictive Internet use, whereas smoking might have acted as a substitution for boys. Boys might have fewer opportunities for smoking because they usually played online games at home or Internet café where adolescent smoking is prohibited. In contrast, cyberspace might provide girls more chances to reinforce the drinking and smoking behaviors against a gender-discriminative social atmosphere for women [3], [48]. Girls might be encouraged to drink and smoke by sharing experiences or information on drinking and smoking with their online peers. Such online interactions may contribute to establishing a favorable norm for smoking and drinking which could lead to offline gatherings in pursuit of drinking or smoking.

Our findings on self-rated academic achievement, physical activities, and psychological status confirm previous studies [17], [22], [35]. Self-rated academic achievement was inversely associated with addictive Internet use, yet the association was stronger in boys than girls. The difference might be attributable to unequal pressure for better academic achievement between genders. In a male dominant society, such as in Asian communities with Confucian backgrounds, parental expectations still focus more on boys with the traditional perspective of men as breadwinners, responsible for earning money for their families. As their academic excellence affects later social and economic positions, boys of low academic achievement may be more stressed than their girl counterparts. This societal atmosphere might induce boys to be addicted to the Internet which provides a hideout from reality [3] or eases their stress with illusory feelings of achievement and self-esteem [54]. The boys addicted to the Internet in this way might waste time for study leading iteratively to poor academic achievement (reverse causality). This study also confirms the past results reporting associations of Internet addiction with depression [17], suicidal behaviors [55], lower self-rated sleep satisfaction [3], and substance use [56].

Several limitations of this study should be noted. Firstly, this study used cross-sectional data for which causal relations cannot be inferred. Secondly, despite survey administration to guarantee the anonymity of the subject online, adolescents might under-report or over-report in a socially desirable manner. Lastly, respondents were sampled among adolescents who were attending schools. Although it was a nationally representative survey and the rate of entering middle and high school in Korea has been above 99%, selection bias might exist due to excluded adolescents who were out of school, absentees, and exceptional children.

In summary, we found several significant associations of addictive Internet use with individual- and school-level factors and gender differences. Our results suggest that preventing adolescents' addictive Internet use at a population level should take into account gender differences and the association factors of family and school contexts.

## Supporting Information

Table S1 **Twenty questionnaires of the simplified Korean Internet Addiction Self-assessment Tool (KS scale).**
(DOCX)
