# Supplementary material for: Addictive Internet Use among Korean Adolescents: A National Survey
Source: PLoS One. 2014 Feb 5;9(2):e87819. doi: 10.1371/journal.pone.0087819 (PMC3914839; doi:10.1371/journal.pone.0087819)
Supplement: Table S1 — Twenty questionnaires of the simplified Korean Internet Addiction Self-assessment Tool (KS scale). (DOCX) [file pone.0087819.s001.docx]

Table S1. Table S1. Twenty questionnaires of the simplified Korean Internet Addiction Self-assessment Tool (KS scale)

Have you ever had experiences as followed?

|  |  | Never | Sometimes yes | Frequently yes | always yes |
| --- | --- | --- | --- | --- | --- |
| Disturbance of adaptive functions | 1. Worse health because of internet use |  |  |  |  |
|  | 2. Headache because of excessive internet use |  |  |  |  |
|  | 3. Not doing what you planned because of internet use |  |  |  |  |
|  | 4. Falling asleep in classes as you were tired because of internet use |  |  |  |  |
|  | 5. Failing of [eyesigh](http://endic.naver.com/search.nhn?query=eyesight)t because of internet use |  |  |  |  |
|  | 6. Not able to resist using internet even you have many things to do |  |  |  |  |
| Positive anticipation | 7. Having more self-confidence when you use internet |  |  |  |  |
| Withdrawal | 8. Being bored and dull if you cannot use internet |  |  |  |  |
|  | 9. Dithering or being nervous if you cannot use internet |  |  |  |  |
|  | 10. Recurring to internet contents even when you do not use internet |  |  |  |  |
|  | 11. Being angry with someone who blocks internet use |  |  |  |  |
| Virtual  interpersonal relationship | 12. People who met in cyberspace treat me better that those who have known in real life |  |  |  |  |
|  | 13. More people in cyberspace recognize me than those in real life |  |  |  |  |
|  | 14. Understanding people in cyberspace more than those in real life |  |  |  |  |
| Deviant behaviors | 15. Being tried to deceive the time of internet use |  |  |  |  |
|  | 16. Spending more money because of internet use |  |  |  |  |
| Tolerance | 17. Wanting more if you stop using internet |  |  |  |  |
|  | 18. Failing to lessen time of internet use |  |  |  |  |
|  | 19. Occurring a thought endlessly that you should lessen internet use |  |  |  |  |
|  | 20. Others said that I use internet too much |  |  |  |  |
